# Interfacial Growth of 420 nm Ultrathin and Dense MOFs for Composite Electrolyte to Reduce Li^+^ Conduction Resistance and Inhibit Lithium Dendrite

**DOI:** 10.1002/advs.76317

**Published:** 2026-07-06

**Authors:** Xinhong Qi, Zhaokai Rui, Shichen Zhang, Yihang Li, Ziyi Xin, Mengjuan Li, Lu Gao, Yuchen He, Xiaobin Jiang, Xiangcun Li, Gaohong He

**Affiliations:** ^1^ State Key Laboratory of Fine Chemicals Frontiers Science Center for Smart Materials Oriented Chemical Engineering School of Chemical Engineering Dalian University of Technology Dalian China

**Keywords:** composite solid electrolytes, ion transport kinetics, lithium dendrite inhibition, lithium metal batteries, ultrathin metal organic frameworks

## Abstract

Metal organic frameworks (MOFs) have attracted increasing attention in composite solid electrolytes (CSEs), attributed to the tunable pore structures and good thermal and electrochemical stability. However, MOFs‐based CSEs still exist, with a trade‐off between ionic conductivity and lithium dendrite inhibition ability. Herein, 420 nm ultrathin ZIF‐67 layer on polyvinylidene fluoride‐co‐hexafluoropropylene (PVDF‐HFP) membrane is precisely regulated through the interfacial growth method. The organic ligand 2‐methylimidazole permeates through the straight‐through pore of PVDF‐HFP to react with Co^2+^, which controls the reaction rate to form ultrathin and dense ZIF‐67. The CSE with ultrathin and dense ZIF‐67 layer not only reduces Li^+^ transport resistance but also promotes lithium salts disassociation, contributing to an ionic conductivity of 2.70 mS cm–^1^ at 30°C and Li^+^ transference number of 0.71. Meanwhile, the ultrathin ZIF‐67 induces fast Li^+^ transport kinetics, uniform Li^+^ flux, and homogeneous Li deposition, synergizing with high mechanical strength to inhibit lithium dendrite growth. As a result, the NCM811||Li coin battery shows improved cycling performance with a capacity retention of 70% after 446 cycles at 0.5 C and is capable of working at −20°C. This work proposes an innovative design of ultrathin MOFs‐based CSEs with high ion conductivity and low interfacial conduction resistance.

## Graphical abstract

In this work, an interfacial growth method using PVDF‐HFP enables controlled permeation of 2‐methylimidazole to react with Co^2+^, forming an ultrathin (420 nm) ZIF‐67 layer by precisely tuning the reaction time. This dense layer promotes lithium salt dissociation and suppresses dendrite growth, while its ultrathin nature reduces Li^+^ transport resistance, achieving high ionic conductivity of 2.70 mS cm^−1^ (30 °C).

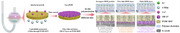

## 1 Introduction

Lithium‐ion batteries (LIBs) have been extensively applied in portable electronics, new energy vehicles, and other important areas attributed to their high energy density, long operational life, and low self‐discharge rate [1, 2, 3]. However, traditional liquid LIBs still face various problems such as poor thermal stability, leakage and combustion of flammable liquid electrolyte as well as ineluctable lithium dendrite growth [4, 5, 6]. With the aggravation of global energy crisis, the demand of energy from power grid and electrical vehicles have rapidly grown. Nevertheless, the energy density of traditional liquid LIB is approaching its theoretical limit, which makes it reluctant to satisfy the increasing demand. Consequently, the pursuit of batteries with higher energy density and greater safety have become considerable challenges for contemporary battery development [7, 8, 9, 10].

Solid‐state batteries (SSBs) have attracted tremendous interest in both academia and industry for the unique advantages, such as non‐flammability and a broad electrochemical stability window by replacing liquid electrolytes with solid‐state electrolytes (SSEs), which enables them to match with high‐capacity electrodes [11, 12, 13]. Meanwhile, lithium metal anode is considered as a candidate material for the anode of SSBs attributed to its high theoretical specific capacity (3860 mAh g^−1^) and a low redox potential (‐3.04 V vs. the standard hydrogen electrode) [14, 15, 16]. Consequently, the solid‐state lithium metal batteries (SSLMBs) are expected to become the candidate for next‐generation energy storage and power batteries [17]. The SSEs contain organic polymer electrolytes, inorganic solid electrolytes, and composite solid electrolytes (CSEs). Although polymer electrolytes exhibit excellent flexibility and processability, the low room‐temperature ionic conductivity, low mechanical strength, and poor electrochemical stability have limited their applications. Inorganic electrolytes demonstrate superior ionic conductivity and mechanical strength, but they suffer from inherent fragility and poor compatibility with electrodes due to the “point‐to‐point contact” between electrolytes and electrodes [18]. CSEs combine the advantages of both organic polymer and inorganic ceramic electrolytes, which are considered as one of the most promising candidates for SSEs in SSLMBs [19, 20].

Metal‐organic frameworks (MOFs) with large specific surface area, tunable pore structures, regular channels, and good thermal and electrochemical stability have shown broad applicability prospects in the field of SSEs [21, 22]. The porous structure of MOFs and the Lewis acid‐base effect between the metal cation in MOFs and the anion in the lithium salt will promote dissociation of the lithium salt, thus accelerating Li^+^ transport [23]. Meanwhile, MOFs have relatively high mechanical strength, which enables them to suppress lithium dendrite growth [24, 25]. In recent years, numerous potential MOFs fillers in SSEs have been investigated. For example, Song et al. have used in‐situ grown ZIF‐67 on 3D cellulose fiber skeleton, succinonitrile (SN) plasticizer, and poly(ethylene oxide) (PEO) to construct ZIF‐67@CF/PEO‐SN CSE. This CSE membrane shows a thickness of 80 µm and enhanced mechanical strength with ionic conductivity of 0.117 mS cm^−1^ at 30°C [26]. Liu et al. have integrated a 5.1 µm in‐situ polymerized quasi‐solid electrolyte layer with Kevlar nanofiber bridged UiO‐66 as a rigid skeleton on the cathode. This constructed topological network demonstrates high rigidity (5.4 GPa) and good ion‐regulated properties, contributing to homogenized Li^+^ flux, excellent dendrite suppression, and high ionic conductivity of 0.73 mS cm^−1^ at room temperature [27]. Guo et al. have proposed an innovative host‐guest in‐situ polymerized MOFs‐gel polymer electrolyte (GPE) with a thickness of approximately 120 µm. The GPE employs Ti‐MOFs with synergistic porous sites serving as the “host–guest” platform to modulate the performance, in which the Ti‐MOFs not only serve as an efficient promoter for Li^+^ conduction (1.36 mS cm^−1^, 25°C), but also exhibit excellent mechanical strength and high‐voltage resistance [28]. An et al. have elucidated Li^+^ conduction behavior in glassy ZIF‐62 GPE (74 µm) at the molecular level, in which Li^+^ migration is accomplished by the continuous delivery of N sites in imidazole and benzimidazole ligands. Such fast Li^+^ transport (0.332 mS cm^−1^, 20°C) increases Li^+^ transference number (0.74) and facilitates the inorganic‐dominated interface generation, thus the long‐term stability with remarkable high‐rate capability was achieved [29]. Xu et al. have developed a glass MOF‐based SSE with a thickness of 200 µm for an all‐solid‐state Li‐O battery. The non‐flammable and boundary‐free glass MOF SSEs are capable of suppressing the dendrite growth and exhibiting long‐term Li plating/stripping stability, contributing to an ionic conductivity of 0.5 mS cm^−1^ at 20°C, a high Li^+^ transference number of 0.86, and good electrochemical stability [30].

However, MOFs‐based CSEs still face a trade‐off between ionic conductivity and lithium dendrite suppression ability. When the thickness of MOFs membrane is increased to improve the mechanical strength and restrain lithium dendrite growth, it simultaneously increases Li^+^ transport resistance due to its intrinsic non‐Li^+^ conductor character. Thus, the Li^+^ transport is impeded, resulting in reduced ionic conductivity. On the contrary, the loose MOFs distribution cannot uniform the Li^+^ flux distribution, which results in uneven lithium deposition and lithium dendrite growth. Furthermore, the reported MOFs‐based CSEs usually employed MOFs fillers to incorporate into the polymer matrix, contributing to the overall MOF distribution along the entire CSEs membrane thickness. As the MOFs distribution in whole CSEs is difficult to thin, which imposes definite resistance for Li^+^ transport, the ionic conductivity is usually smaller than 1.5 mS cm^−1^. Consequently, it is necessary to develop thin MOFs decorated CSEs with novel microstructures that can precisely balance ionic conductivity and effective dendrite suppression ability.

**FIGURE 1:**
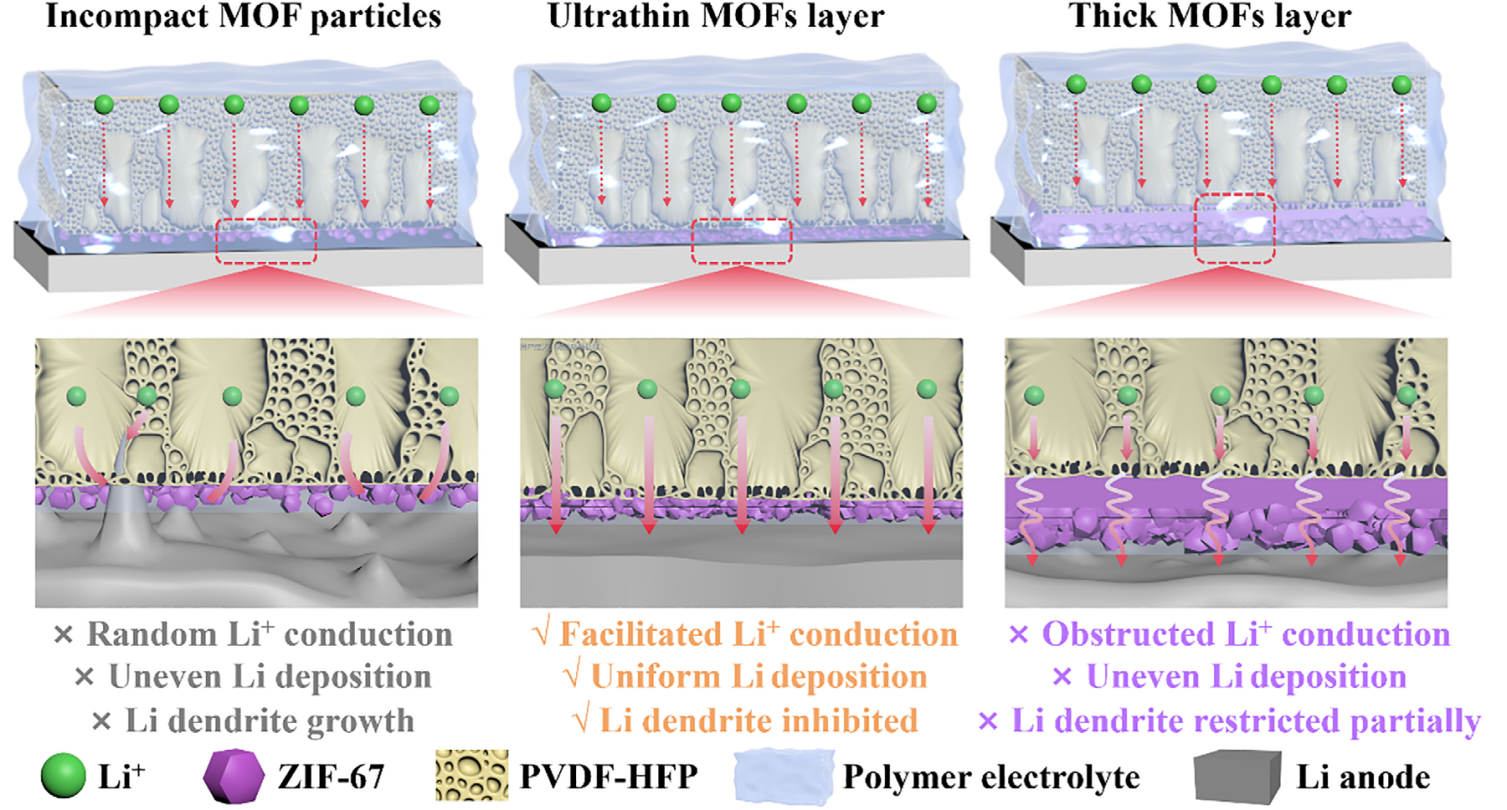
Schematic illustration of an ultrathin ZIF‐67 layer to promote uniform Li^+^ conduction and inhibit lithium dendrite growth.

Herein, a series of novel ultrathin (250 nm, 420 nm) and dense ZIF‐67‐based CSEs are designed to reduce Li^+^ conduction resistance due to the ultrathin peculiarity and induce uniform Li deposition with the dense morphology, thereby balancing the trade‐off between ionic conductivity and lithium dendrite inhibition ability as shown in Figure 1. The ultrathin and dense ZIF‐67 on PVDF‐HFP membrane is precisely regulated through the interfacial growth method, during which the ligand 2‐methylimidazole (2‐MIm) is controllably permeated through the straight‐through pores of PVDF‐HFP to react with metal ion Co^2+^. The ultrathin and dense ZIF‐67 on PVDF‐HFP is named as P_n_Z_m_ (n is the thickness of the membrane in µm, m is the interfacial growth time in minutes) and employed as a base membrane to support in‐situ polymerized electrolyte to construct P_n_Z_m_PEDF CSEs (the electrolyte precursor PEDF represents the composition of the precursor solution, including PEGDA, EC, DMC, FAN, LiTFSI, and LiDFOB). The vertically aligned highways and ultrathin ZIF‐67 layer are proposed to decrease Li^+^ conduction resistance. And more free Li^+^ is liberated via Lewis acid‐base effect and confinement of ZIF‐67 with anions, thus a high ionic conductivity of 2.70 mS cm^−1^ at 30°C and Li^+^ transference number of 0.71 are achieved by P_15_Z_30_PEDF (420 nm ZIF‐67 layer). In addition, the P_15_Z_30_PEDF exhibits improved mechanical strength and uniform Li^+^ flux to restrain lithium dendrite growth. As a result, the Li|P_15_Z_30_PEDF|Li symmetric cell demonstrates a highly stable lithium deposition/stripping performance for 1000 h at 0.1 mA cm^−2^. The NCM811|P_15_Z_30_PEDF|Li coin battery shows improved rate and cycling performance with a capacity retention of 70% after 480 cycles at 0.5 C, and is capable of operating at −20°C–60°C. The corresponding pouch cell exhibits a capacity retention of 82.5% after 100 cycles at 0.1 C.

## 2 Results and Discussion

**FIGURE 2:**
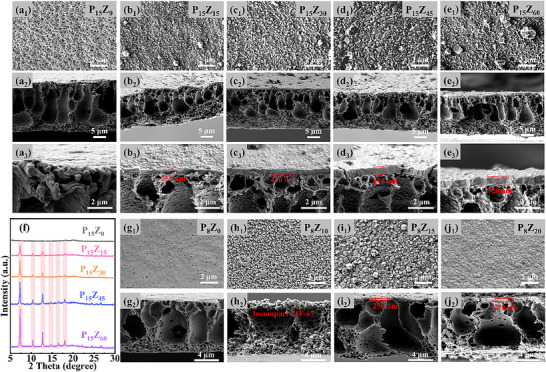
Synthesis and characterization of ultrathin MOF on PVDF‐HFP membranes. SEM images of surface and cross section for (a_1_‐a_3_) P_15_Z_0_, (b_1_‐b_3_) P_15_Z_15_, (c_1_‐c_3_) P_15_Z_30_, (d_1_‐d_3_) P_15_Z_45_, (e_1_‐e_3_) P_15_Z_60_; (f) XRD patterns of P_15_Z_m_ membranes; SEM images of surface and cross section for (g_1_‐g_2_) P_8_Z_0_, (h_1_‐h_2_) P_8_Z_10_, (i_1_‐i_2_) P_8_Z_15_, (j_1_‐j_2_) P_8_Z_20_ membranes.

The ultrathin and dense ZIF‐67 on PVDF‐HFP membrane is precisely synthesized through interfacial reaction of the straight‐through pore controllably permeated 2‐MIm with Co^2+^ on the finger‐like pore side of PVDF‐HFP membrane, as shown in Figure 2a_1_–a_3_. The self‐made membrane preparation device is shown in Figure S1, in which the PVDF‐HFP membrane is used to separate Co^2+^ solution and 2‐MIm solution. The PVDF‐HFP membrane with finger‐like straight‐through pore could control the 2‐MIm permeation through the membrane via capillarity, and then the permeated 2‐MIm reacts with Co^2+^ on PVDF‐HFP membrane surface. The controllably permeated 2‐MIm would reduce the reaction rate of Co^2+^ with 2‐MIm to form an ultrathin ZIF‐67 layer, rather than the traditional solution method with Co^2+^ and 2‐MIm reaction directly to result in explosive growth of ZIF‐67 and thick layer formation. The scanning electron microscopy (SEM) images of PVDF‐HFP membrane in Figure 2a_1_–a_3_ demonstrate the finger‐like pore throughout the membrane and the porous surface, which could control the permeation of 2‐MIm to slow down its reaction rate with Co^2+^, thus providing a prerequisite for the ultrathin MOF layer formation. The SEM images in Figure 2b_1_–e_1_ verify the dense ZIF‐67 layer on P_15_Z_m_ membranes. And the cross‐sectional views in Figure 2b_2_–e_2_ confirm the finger‐like straight‐through pore in P_15_Z_m_, which also reduces Li^+^ conduction resistance to improve the ionic conductivity [31]. More importantly, SEM images have clearly revealed that the ZIF‐67 nanoparticles tend to grow on the straight‐through porous side and gradually form a dense layer during the interfacial growth reaction. The thickness of the ZIF‐67 layer progressively increases as the reaction time is prolonged, as clearly depicted in Figure 2b_3_–e_3_. To be specific, the thickness of the ZIF‐67 dense layer of P_15_Z_15_, P_15_Z_30_, P_15_Z_45_, and P_15_Z_60_ is 350 nm, 420 nm, 610 nm, and 720 nm, respectively. Thus, the thickness of the ZIF‐67 layer has been precisely regulated through interfacial reaction time. The cross‐sectional EDS mapping (Figure S2) and cross‐sectional TEM images (Figure S3) also demonstrate the successful formation of the ZIF‐67 layer. These dense ZIF‐67 layers with controllable thickness would regulate Li^+^ conduction resistance and lithium dendrite inhibition ability, thereby enhancing the cycle stability of the battery [24]. The X‐ray diffraction (XRD) was applied to prove the growth of ZIF‐67 on P_15_Z_m_ membranes, as shown in Figure 2f. The significant diffraction peaks at 2θ = 7.5° and specific peaks at 2θ = 10.4°, 12.7°, 14.7°, 16.5° and 18.0° demonstrate the successful synthesis and growth of ZIF‐67 layer on P_15_Z_m_ membranes [26, 32]. Moreover, the membrane and ZIF‐67 layer could be further thinned by adjusting the precursor solution concentration and the interfacial growth time. The decrease in ZIF‐67 layer thickness is attributed to the lower resistance to diffusion of 2‐MIm under the concentration gradient in thinner base membranes, which leads to the formation of more ZIF‐67 nuclei during the nucleation stage [33]. During the subsequent growth process, these nuclei rapidly form a dense ZIF‐67 layer, thereby retarding the further growth rate and resulting in a thinner ZIF‐67 layer. The P_8_Z_0_ membrane with a thickness of 8 µm in Figure 2g_1_–g_2_ could further decrease the thickness of the dense ZIF‐67 layer. The SEM images in Figure 2h_1_–h_2_ demonstrate that the ZIF‐67 particles are randomly dispersed on the surface of the P_8_Z_10_ membrane without forming a dense ZIF‐67 layer. This is attributed to the fact that the thin membrane could not control the permeation of 2‐MIm precisely. Interestingly, the dense ZIF‐67 layers with thinner thicknesses of 250 nm (Figure 2i_1_–i_2_) and 470 nm (Figure 2j_1_–j_2_) are further obtained on P_8_Z_15_ and P_8_Z_20_ membranes, respectively. The dense and thin ZIF‐67 layer is formed by the makeup growth within the voids of the loose ZIF‐67 nanoparticles as the interfacial growth time increases.

After that, the electrolyte precursor was added onto P_15_Z_m_ membranes, followed by thermal treatment to fabricate P_15_Z_m_PEDF electrolytes. The photograph in Figure S5a exhibits the liquid electrolyte precursor before polymerization, which clearly polymerizes into a solid state after thermal treatment, as shown in Figure S5b,c. Furthermore, the Fourier transform infrared (FTIR) spectra of P_15_Z_m_PEDF CSEs in Figure S6 demonstrate that the C═C stretching vibration peaks in the electrolyte precursor at 1600–1640 cm^−1^ disappear, which certifies the successful polymerization PEGDA [34]. The thermogravimetric analysis (TGA) curves of P_15_Z_0_PEDF, P_15_Z_15_PEDF, P_15_Z_30_PEDF, P_15_Z_45_PEDF, and P_15_Z_60_PEDF are exhibited in Figure S7. The first weight loss stage at 150°C results from the decomposition and volatilization of plasticizers (EC, DMC, FAN) [35]. The second weight loss stage at 400°C is mainly due to the decomposition of PVDF‐HFP and LiTFSI. As a result, the weight loss has a downward trend with the increase of ZIF‐67 layer thickness, which is attributed to the ZIF‐67 adsorption and confinement of the electrolyte to enhance the thermal stability.

**FIGURE 3:**
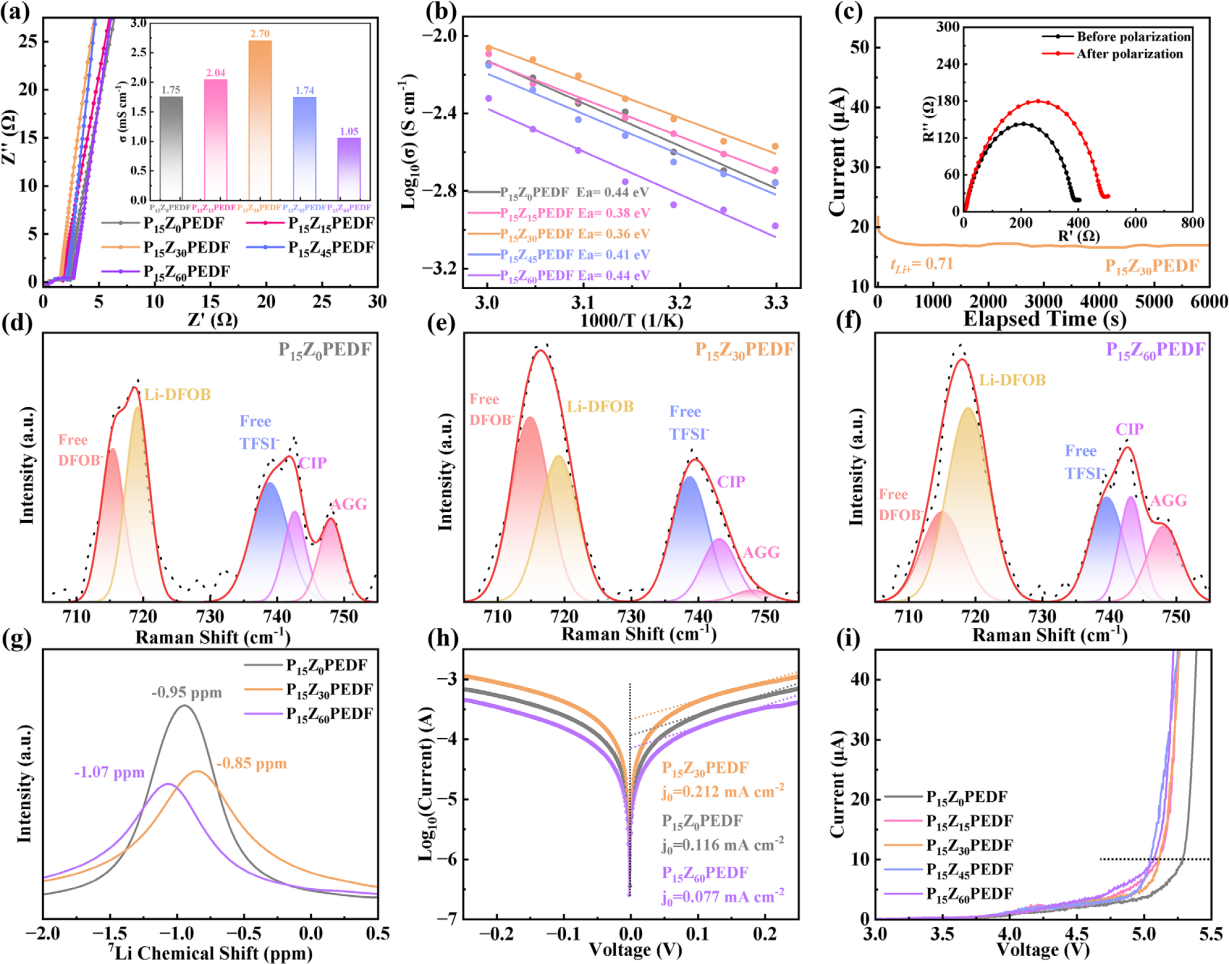
Li^+^ transport and electrochemical stability of CSEs. (a) EIS curves and ionic conductivities of P_15_Z_m_PEDF at 30°C; (b) Arrhenius plots from 30 to 60°C of P_15_Z_m_PEDF and the activation energies; (c) Direct current polarization curve with EIS curves before and after polarization of Li|P_15_Z_30_PEDF|Li cell; The Raman spectra of (d) P_15_Z_0_PEDF, (e) P_15_Z_30_PEDF and (f) P_15_Z_60_PEDF; (g) ^7^Li MAS NMR spectra of P_15_Z_m_PEDF; (h) Tafel curves of P_15_Z_m_PEDF; (i) LSV curves of P_15_Z_m_PEDF.

As a basic parameter for CSEs, ionic conductivities of P_15_Z_0_PEDF, P_15_Z_15_PEDF, P_15_Z_30_PEDF, P_15_Z_45_PEDF, and P_15_Z_60_PEDF at different temperatures were tested and calculated based on the electrochemical impedance spectroscopy (EIS) plots from 30 to 60°C in Figure S8. As clearly depicted in Figure 3a, the P_15_Z_30_PEDF exhibits lower electrochemical impedance and possesses the highest ionic conductivity of 2.70 mS cm^−1^ at 30°C. While the P_15_Z_0_PEDF, P_15_Z_15_PEDF, P_15_Z_45_PEDF, and P_15_Z_60_PEDF show lower ionic conductivities of 1.75 mS cm^−1^, 2.04 mS cm^−1^, 1.74 mS cm^−1^, and 1.05 mS cm^−1^ at 30°C, respectively. The ionic conductivity reaches its peak at a growth time of 30 min, thus P_15_Z_30_PEDF is selected as the experimental sample, while P_15_Z_0_PEDF and P_15_Z_60_PEDF are control samples for further tests. Meanwhile, the ionic conductivity of P_8_Z_m_ electrolytes is exhibited in Figure S9, and the P_8_Z_15_PEDF demonstrates the highest ionic conductivity of 1.46 mS cm^−1^ at 30°C. Moreover, the logarithm of ionic conductivities versus the reciprocal of Kelvin temperatures is plotted in Figure 3b to explore the Li^+^ transference activation energies of P_15_Z_m_PEDF electrolytes, and the corresponding activation energies are calculated through the Arrhenius equation. As a result, the P_15_Z_30_PEDF demonstrates the lowest activation energy of 0.36 eV, indicating the lowest Li^+^ transference energy barrier among P_15_Z_0_PEDF, P_15_Z_15_PEDF, P_15_Z_45_PEDF, and P_15_Z_60_PEDF with higher activation energies of 0.44 eV, 0.38 eV, 0.41 eV, and 0.44 eV. After adding ZIF‐67, the ZIF‐67 nanoparticles can disrupt the chain arrangement of the PVDF‐HFP polymer and reduce its crystallinity. As a result, lithium ions migrate primarily along the amorphous regions, and the reduced crystallinity enhances segmental motion. Additionally, porous structure along with Lewis acid–base interactions between ZIF‐67 and TFSI^−^ promote the dissociation of lithium salt and enhance Li^+^ transport. However, when the amount of ZIF‐67 is excessive, the ZIF‐67 particles tend to aggregate, which impedes the ion transport pathways and consequently increases the activation energy required for lithium‐ion conduction [36, 37]. After that, the Li^+^ transference number of P_15_Z_m_PEDF CSEs was calculated by the Bruce‐Vincent equation through direct current (DC) polarization curve and EIS plots before and after polymerization, as depicted in Figure 3c and Figure S10. The Li^+^ transference numbers of P_15_Z_0_PEDF, P_15_Z_15_PEDF, P_15_Z_30_PEDF, P_15_Z_45_PEDF, and P_15_Z_60_PEDF are 0.21, 0.56, 0.71, 0.42, and 0.38, respectively. Among these, the P_15_Z_30_PEDF shows the highest Li^+^ transference number, which helps to slow the nucleation time of lithium deposition on the lithium metal anode (LMA), thus improving the cycling stability and lifetime of the battery [26]. Conclusively, the P_15_Z_30_PEDF with 420 nm ultrathin and dense ZIF‐67 layer shows enhanced ionic conductivity, Li^+^ transference number, and decreased activation energy than P_15_Z_0_PEDF and P_15_Z_15_PEDF. This is attributed to the ZIF‐67 layer, which could facilitate lithium salt dissociation by porous structure and Lewis acid‐base interaction. However, when the thickness of the ZIF‐67 layer is increased to 610 nm in P_15_Z_45_PEDF and 720 nm in P_15_Z_60_PEDF, the Li^+^ transport is impeded due to the increased resistance. Therefore, the P_15_Z_0_PEDF, P_15_Z_30_PEDF, and P_15_Z_60_PEDF are further investigated to clarify the intrinsic mechanism of fast Li^+^ transport kinetics and the cell performance.

The coordination states of Li^+^ with anions were emphasized through Raman spectroscopy as shown in Figure 3d–f and Figure S11. To be specific, the peaks with Raman shift at 719 cm^−1^ and 714 cm^−1^ are Li‐DFOB and free DFOB^−^, respectively [38]. Whereas the peaks at Raman shift of 748 cm^−1^, 743 cm^−1^, 739 cm^−1^ reveal the aggregated anions (AGG, two or more TFSI^−^ coordinate with one or more Li^+^), coordinated ion pair (CIP, one TFSI^−^ coordinates with one Li^+^), and free TFSI^−^, respectively [39, 40]. The contents of free TFSI^−^ and free DFOB^−^ are 63.9% and 55.7% in P_15_Z_30_PEDF, which are significantly higher than those for P_15_Z_0_PEDF (52.1% free TFSI^−^, 43.6% free DFOB^−^) and P_15_Z_60_PEDF (41.4% free TFSI^−^, 31.4% free DFOB^−^) as shown in Figure S7. This demonstrates more dissociation of lithium salts in P_15_Z_30_PEDF to release free Li^+^. Furthermore, the high‐resolution ^7^Li magic angle spinning nuclear magnetic resonance (MAS NMR) was applied to characterize the chemical environments around Li^+^. As shown in Figure 3g, the chemical shift of P_15_Z_30_PEDF moves downfield (−0.85 ppm) compared with P_15_Z_0_PEDF (−0.95 ppm) and P_15_Z_60_PEDF (−1.07 ppm). This downfield chemical shift indicates the reduced shielding effect around Li^+^ in P_15_Z_30_PEDF, exhibiting the weaker coordination of Li^+^ with anions. This further proves the greater dissociation of lithium salts in P_15_Z_30_PEDF. The increased lithium salt disassociation ability of P_15_Z_30_PEDF results from the 3.4 Å and 11.6 Å pore sizes of ZIF‐67 [41], which allow Li^+^ (0.76 Å) to pass through smoothly while restricting the movement of the larger DFOB^−^ (7.36 Å) and TFSI^−^ (7.9 Å) [42]. Additionally, the Lewis acid‐base interaction between ZIF‐67 and anions (DFOB^−^ and TFSI^−^) further limits the anions' mobility, thereby promoting lithium salt dissociation and enhancing Li^+^ migration [22]. On the other hand, the thick and dense ZIF‐67 layer in P_15_Z_60_PEDF exerts high resistance on the Li^+^ transport channel, thus hindering the disassociation of the lithium salt and exhibiting a low Li^+^ transference number accompanied by an upfield chemical shift in ^7^Li NMR.

After that, the exchange current densities of P_15_Z_0_PEDF, P_15_Z_30_PEDF, and P_15_Z_60_PEDF were calculated through fitting Tafel curves from 0.12 V to 0.15 V. As depicted in Figure 3h, the exchange current density of P_15_Z_30_PEDF is 0.212 mA cm^−2^, which is 1.83 times that of P_15_Z_0_PEDF (0.116 mA cm^−2^) and 2.75 times that of P_15_Z_60_PEDF (0.077 mA cm^−2^). The significantly enhanced exchange current density of P_15_Z_30_PEDF indicates faster interfacial Li^+^ transport kinetics than P_15_Z_0_PEDF and P_15_Z_60_PEDF. The electrochemical stability of the electrolyte is essential for coupling with a high‐voltage cathode. Therefore, the linear scanning voltammetry (LSV) curves of P_15_Z_m_PEDF are illustrated in Figure 3i. The electrochemical stability window (ESW) is determined by the voltage corresponding to 10 µA for the potential at a current density of 5 µA cm^−2^, which is recognized as the initial decomposition voltage [43]. The ESW of P_15_Z_0_PEDF, P_15_Z_15_PEDF, P_15_Z_30_PEDF, P_15_Z_45_PEDF, and P_15_Z_60_PEDF are 5.28 V, 5.11 V, 5.11 V, 5.04 V, and 5.06 V, respectively. Both P_15_Z_m_PEDF CSEs demonstrate the ESW over 5.0 V, which enables them to match with high voltage cathode like LiNi_0.8_Co_0.1_Mn_0.1_O_2_ (NCM811). The ESW of P_15_Z_30_PEDF is a bit lower than P_15_Z_0_PEDF, which might result from the porous ZIF‐67 nanoparticle having a higher affinity with plasticizer molecules with relatively low resistance to oxidation [44]. Thus, the P_15_Z_30_PEDF with 420 nm ultrathin and dense ZIF‐67 layer shows enhanced Li^+^ transport kinetics and excellent antioxidation ability, which is expected to improve the battery performance.

**FIGURE 4:**
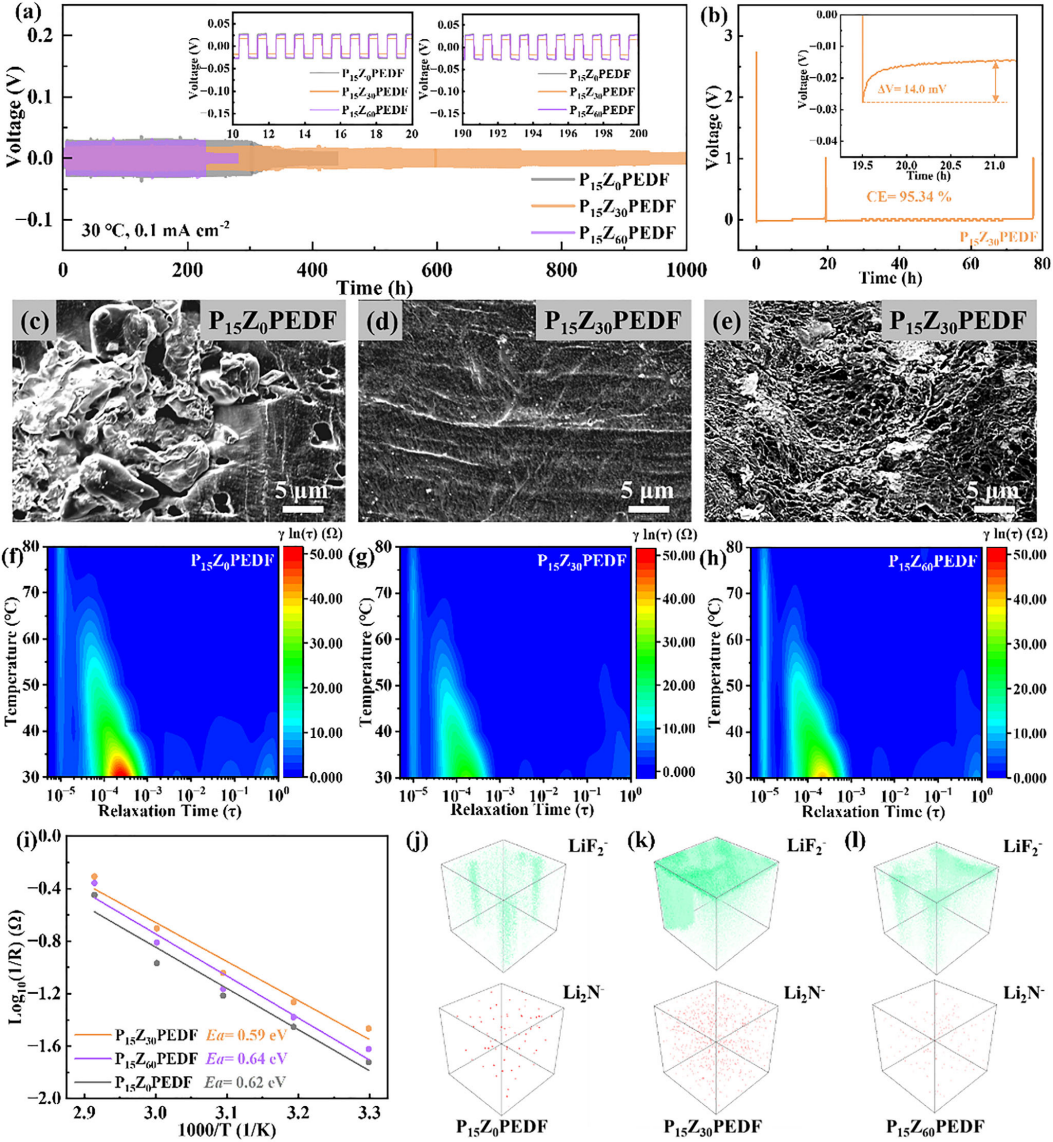
Interfacial stability towards LMA. (a) Cycling performances of Li|P_15_Z_m_PEDF|Li at 0.1 mA cm^−2^; (b) Nucleation overpotential and CE of Li|P_15_Z_30_PEDF|Cu; SEM images of LMA disassembled from (c) Li|P_15_Z_0_PEDF|Li, (d) Li|P_15_Z_30_PEDF|Li and (e) Li|P_15_Z_60_PEDF|Li after 50 h cycling at 0.1 mA cm^−2^; DRT intensity color map of in‐situ impedance at 30 to 80°C for (f) Li|P_15_Z_0_PEDF|Li, (g) Li|P_15_Z_30_PEDF|Li, (h) Li|P_15_Z_60_PEDF|Li; The interfacial activation energy of Li|P_15_Z_m_PEDF|Li; TOF‐SIMS 3D images of LiF_2_^−^ and Li_2_N^−^ for LMA disassembled from (j) Li|P_15_Z_0_PEDF|Li, (k) Li|P_15_Z_30_PEDF|Li, (l) Li|P_15_Z_60_PEDF|Li after 50 h cycling at 0.1 mA cm^−2^.

The Li|P_15_Z_0_PEDF|Li, Li|P_15_Z_30_PEDF|Li, and Li|P_15_Z_60_PEDF|Li symmetric cells were fabricated and operated to evaluate the interfacial stability between CSEs and LMA. The long‐term cycling performances of Li||Li symmetric cells are illustrated in Figure 4a, in which the Li|P_15_Z_30_PEDF|Li demonstrates stable cycling for 1000 h at a current density of 0.1 mA cm^−2^, while the Li|P_15_Z_0_PEDF|Li and Li|P_15_Z_60_PEDF|Li face short circuit after 333 h and 229 h, respectively. Meanwhile, the Li|P_15_Z_30_PEDF|Li battery can stably cycle for over 140 h and 113 h at current densities of 0.2 and 0.3 mA cm^−2^, respectively. The critical current density reaches 3.5 mA cm^−2^ as depicted in Figure S12. The smaller initial overpotential of Li|P_15_Z_30_PEDF|Li (0.017 V) compared with Li|P_15_Z_0_PEDF|Li (0.028 V) and Li|P_15_Z_60_PEDF|Li (0.025 V) indicates a lower interfacial impedance of Li|P_15_Z_30_PEDF|Li. To further evaluate the Li deposition process and Li utilization rate, the Li|P_15_Z_0_PEDF|Cu, Li|P_15_Z_30_PEDF|Cu, and Li|P_15_Z_60_PEDF|Cu cells were assembled to measure the Li nucleation overpotential and the coulombic efficiency (CE) through the previously reported method [45]. The nucleation overpotential of Li|P_15_Z_30_PEDF|Cu has decreased to 14 mV (Figure 4b), which is lower than that for Li|P_15_Z_0_PEDF|Cu (16.5 mV, Figure S13a) and Li|P_15_Z_60_PEDF|Cu (17.0 mV, Figure S13b). The decreased nucleation overpotential is attributed to the fact that the ultrathin ZIF‐67 layer in P_15_Z_30_PEDF and the enhanced Li^+^ transport kinetics distribute Li^+^ flux uniformly. This assists Li uniform nucleation, which is beneficial for stable Li plating/stripping and leads to high CE of Li|P_15_Z_30_PEDF|Cu cell. Thus, the Li|P_15_Z_30_PEDF|Cu cell exhibits a higher average CE of 95.34% than Li|P_15_Z_0_PEDF|Cu of 91.58% and Li|P_15_Z_60_PEDF|Cu of 94.07%, as shown in Figure S13 and Figure 4b.

The SEM images of LMA disassembled from Li||Li symmetric cells are demonstrated in Figures 4c–e to visually demonstrate the interface changes during cycling. As clearly exhibited in Figure 4c, dramatic lithium dendrite growth is detected on LMA disassembled from Li|P_15_Z_0_PEDF|Li after 50 h cycling at 0.1 mA cm^−2^, which results from the sluggish Li^+^ transport and uneven Li deposition. On the contrary, LMA from cycled Li|P_15_Z_30_PEDF|Li is basically smooth (Figure 4d), confirming the ultrathin and dense ZIF‐67 layer promoted uniform Li deposition and inhibited lithium dendrite growth. Though P_15_Z_60_PEDF has a relatively thick and dense ZIF‐67 layer, enabling it to suppress lithium dendrite growth to some degree, the unfavorable Li^+^ transport and uneven Li deposition still result in tiny dendrite growth (Figure 4e). Meanwhile, the Young's modules of P_15_Z_0_PEDF, P_15_Z_30_PEDF, and P_15_Z_60_PEDF were detected by atomic force microscope (AFM) to evaluate the mechanical strength, as shown in Figure S14. The ultrathin and dense ZIF‐67 layer significantly increases the Young's modules of P_15_Z_30_PEDF (3.75 GPa) and P_15_Z_60_PEDF (4.48 GPa), which are much higher than that of P_15_Z_0_PEDF (629 MPa). The enhanced mechanical strength with ultrathin and dense ZIF‐67 layer could also effectively inhibit lithium dendrite growth, which is coordinated with the SEM images [46].

To further verify the interfacial stability towards LMA, the EIS plots of Li|P_15_Z_0_PEDF|Li, Li|P_15_Z_30_PEDF|Li, and Li|P_15_Z_60_PEDF|Li from 30 to 80°C were measured. To be specific, the impedance changes associated with Li^+^ migration and conversion at different temperatures were decoupled through the impedance and related distribution of relaxation time (DRT) analyses. The peaks at 10^−6^–10^−5^ s and 10^−4^–10^−3^ s represent the bulk resistance (*R_b_*) and interfacial resistance (*R_i_*), respectively [47, 48]. The Li|P_15_Z_30_PEDF|Li demonstrates lower *R_i_* than Li|P_15_Z_0_PEDF|Li and Li|P_15_Z_60_PEDF|Li at each temperature, as clearly depicted in Figure 4f–h, indicating the decreased interfacial Li^+^ transport resistance. Furthermore, the activation energy of interfacial Li^+^ migration was calculated as shown in Figure 4i. The Li|P_15_Z_30_PEDF|Li symmetric cell exhibits the activation energy of 0.59 eV for interfacial Li^+^ migration, which is lower than that for Li|P_15_Z_0_PEDF|Li (0.62 eV) and Li|P_15_Z_60_PEDF|Li (0.64 eV). The decreased interfacial migration activation energy verifies the facilitated Li^+^ transport at the interface and thus inhibits lithium dendrite growth. Moreover, the EIS profiles of Li|P_15_Z_0_PEDF|Li, Li|P_15_Z_30_PEDF|Li, and Li|P_15_Z_60_PEDF|Li before cycling and after 10 h, 20 h cycling at 0.1 mA cm^−2^ are depicted in Figure S15, in which the Li|P_15_Z_30_PEDF|Li exhibits the smallest interfacial impedance before and after cycling. Thus, the Li|P_15_Z_30_PEDF|Li symmetric cell shows low interfacial resistance.

Aside from the mechanical strength and Li deposition behavior, the SEI compositions were widely recognized to exert an important influence on interfacial Li^+^ transport kinetics. The deep etching X‐ray photoelectron spectroscopy (XPS) and time‐of‐flight secondary ion mass spectrometry (TOF‐SIMS) were applied on LMA disassembled from Li|P_15_Z_0_PEDF|Li, Li|P_15_Z_30_PEDF|Li, and Li|P_15_Z_60_PEDF|Li symmetric cells after cycling for 50 h to identify the SEI compositions. The deep etching XPS spectra of C 1s are exhibited in Figure S16, the C─C, C─O/C─N, C═O/─CN and C─F peaks at 284.8 eV, 286.5 eV, 288.8 eV and 292.8 eV come from plasticizers and polymer electrolyte [49, 50, 51], and the specific peak at 290.0 eV is attributed to Li_2_CO_3_ formation [52]. The cycled LMA matched with P_15_Z_30_PEDF demonstrates a larger Li_2_CO_3_ content at the surface and 30 s etching, which indicates a thin Li_2_CO_3_ layer concentrated near the SEI surface. As for F 1s spectra in Figure S17, the specific peaks at 688.4 eV, 686.2 eV, and 684.8 eV represent C─F, B─F, and LiF, respectively [53]. In comparison, the cycled LMA using P_15_Z_30_PEDF possesses the largest proportion of LiF at the LMA surface and after etching, which effectively promotes the uniform Li deposition [54]. Furthermore, the N 1s spectra demonstrate the ─C≡N, C─N, Li_3_N and Li_x_N_y_ with specific peaks at 400.8 eV, 399.5 eV, 398.7 eV, 397 eV as shown in Figure S18, respectively [49, 55, 56]. The cycled LMA using P_15_Z_30_PEDF also holds more surface Li_3_N than that with P_15_Z_0_PEDF and P_15_Z_60_PEDF. The more LiF, Li_3_N may be generated from the decomposition of TFSI^−^, DFOB^−^, and FAN absorbed by ZIF‐67 due to the porous structure and Lewis acid‐base effect [57]. The TOF‐SIMS depth profiles (Figure S19) and 3D images (Figures 4j‐l) also confirm that the SEI on cycled LMA from Li|P_15_Z_30_PEDF|Li cell contains a larger amount of LiF_2_^−^ and Li_2_N^−^. The inorganic‐rich SEI in Li|P_15_Z_30_PEDF|Li facilitates interfacial Li^+^ transport and uniform Li deposition, thereby inhibiting lithium dendrite growth [58, 59]. What's more, the 250 nm ultrathin ZIF‐67 also contributes to the long‐term cycling performance of Li||Li symmetric cell. As shown in Figure S20, the Li|P_8_Z_15_PEDF|Li demonstrates stable cycling for 450 h at 0.1 mA cm^−2^, which is more stable than the Li|P_8_Z_0_PEDF|Li, Li|P_8_Z_10_PEDF|Li, and Li|P_8_Z_30_PEDF|Li with short circuit times of 165 h, 163 h, and 50 h, respectively.

**FIGURE 5:**
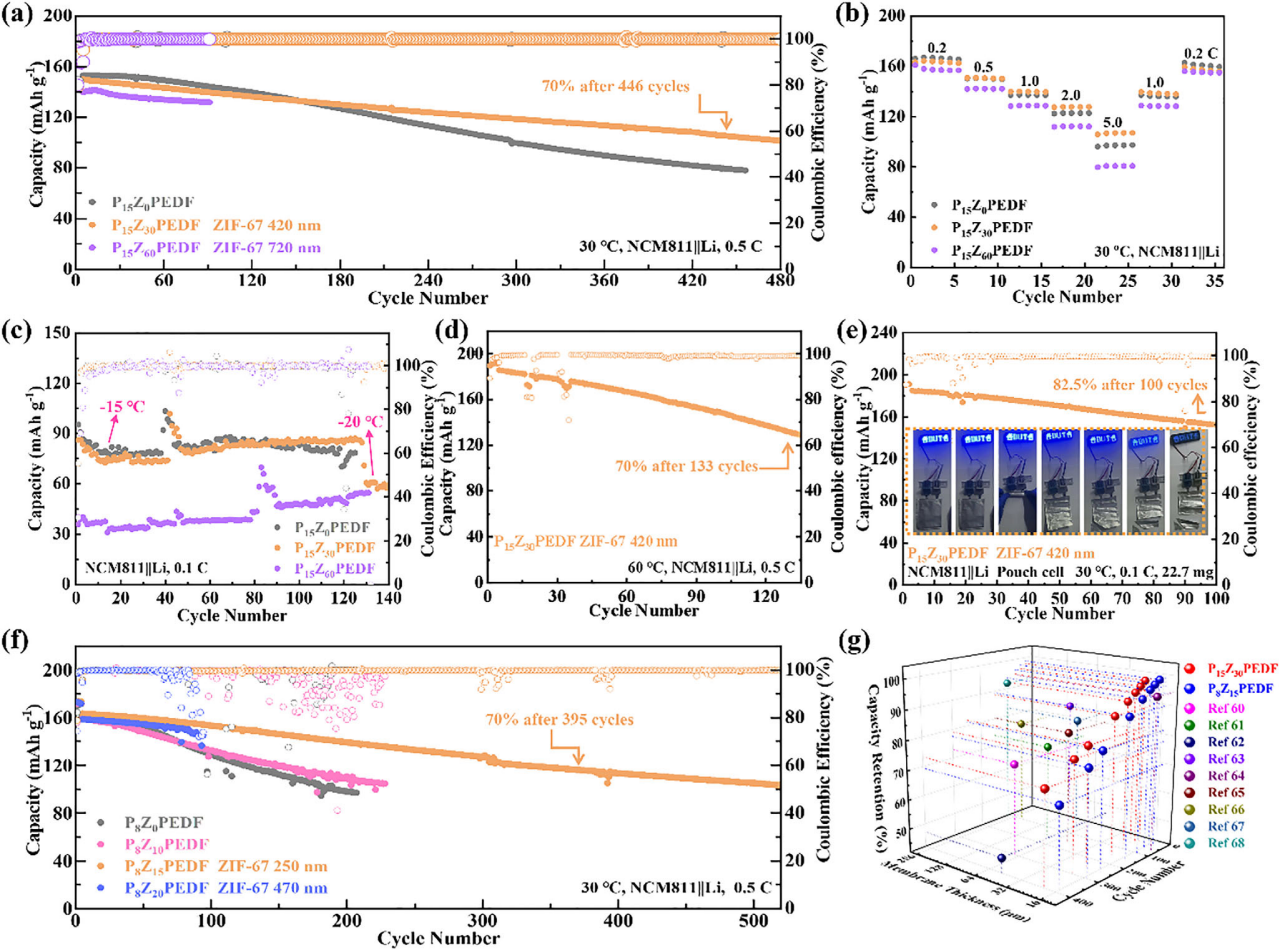
Full cell performance. (a) Cycling performance of NCM811|P_15_Z_m_PEDF|Li at 0.5 C; (b) Rate performance of NCM811|P_15_Z_m_PEDF|Li from 0.2 to 5.0 C; (c) Cycling performance of NCM811|P_15_Z_m_PEDF|Li at −15°C, −20°C; (d) Cycling performance of NCM811|P_15_Z_m_PEDF|Li at 60°C; (e) Cycling performance of NCM811|P_15_Z_30_PEDF|Li pouch cell and corresponding photographs of lighting up LED; (f) Cycling performance of NCM811|P_8_Z_m_PEDF|Li at 0.5 C; (g) Comparisons of cycling performances and electrolyte thickness with other MOFs electrolytes.

To validate the antioxidant feasibility of P_15_Z_m_PEDF toward high‐voltage cathode, the NCM811||Li batteries were assembled to measure the performance under different charge‐discharge rates and different temperatures. Figure 5a exhibits the long‐term cycling performances of NCM811|P_15_Z_m_PEDF|Li at 0.5 C after activation under 0.2 C for 3 cycles at 30°C, and the corresponding charge‐discharge voltage profiles are depicted in Figure S21. The NCM811|P_15_Z_30_PEDF|Li exhibits the specific discharge capacity of 150 mAh g^−1^ and maintains the capacity retention of 70% after 446 cycles. As a contrast, the specific discharge capacity of NCM811|P_15_Z_0_PEDF|Li is 153 mAh g^−1^ and rapidly drops below 70% within 270 cycles. Meanwhile, the NCM811|P_15_Z_60_PEDF|Li possesses a low initial specific discharge capacity of 139 mAh g^−1^ and quickly short‐circuits at the 91^st^ cycle, which might result from the high resistance due to the thick and dense ZIF‐67 layer. Moreover, the rate performances of NCM811|P_15_Z_0_PEDF|Li, NCM811|P_15_Z_30_PEDF|Li and NCM811|P_15_Z_60_PEDF|Li are depicted in Figure 5b. The specific discharge capacities of NCM811|P_15_Z_30_PEDF|Li are 162.6 mAh g^−1^, 150.2 mAh g^−1^, 139.6 mAh g^−1^, 127.7 mAh g^−1^, and 107.1 mAh g^−1^ at 0.2 C, 0.5 C, 1.0 C, 2.0 C, and 5.0 C, respectively. And the discharge capacities are recovered to 137.9 mAh g^−1^ and 159.4 mAh g^−1^ when the current density is returned to 1.0 C and 0.2 C. While the specific discharge capacities of NCM811|P_15_Z_0_PEDF|Li are 165.4 mAh g^−1^, 149.9 mAh g^−1^, 137.1 mAh g^−1^, 122.8 mAh g^−1^, 97.4 mAh g^−1^, 135.8 mAh g^−1^, and 162.8 mAh g^−1^ at 0.2 C, 0.5 C, 1.0 C, 2.0 C, 5.0 C, 1.0 C, and 0.2 C. Even worse, NCM811|P_15_Z_60_PEDF|Li shows more decreased specific discharge capacities of 156.8 mA h g^−1^, 142.1 mA h g^−1^, 128.7 mA h g^−1^, 112.2 mA h g^−1^, 80.8 mA h g^−1^, 128.3 mA h g^−1^, and 156.1 mA h g^−1^ at 0.2 C, 0.5 C, 1.0 C, 2.0 C, 5.0 C, 1.0 C, and 0.2 C. By comparison, the NCM811|P_15_Z_30_PEDF|Li exhibits improved specific discharge capacity than NCM811|P_15_Z_60_PEDF|Li at each rate. Though NCM811|P_15_Z_30_PEDF|Li demonstrates a bit lower capacity than NCM811|P_15_Z_0_PEDF|Li at 0.2 C, the higher specific capacity at high rates still enables P_15_Z_30_PEDF to be a superior choice for high‐rate fast‐charging batteries. The issue of battery failure in low‐temperature environments has long been a concern. Additionally, the battery performance in a chilly environment is essential; the NCM811|P_15_Z_0_PEDF|Li, NCM811|P_15_Z_30_PEDF|Li, and NCM811|P_15_Z_60_PEDF|Li batteries were charged and discharged at −15°C followed by cooling to −20°C under 0.1 C with activation at 0.05 C for 5 cycles as shown in Figure 5c. The NCM811|P_15_Z_0_PEDF|Li exhibits low specific discharge capacity of 81.6 mAh g^−1^ with 78.4 mAh g^−1^ retention after 125 cycles at −15°C and fails at −20°C in a rapid sequence. The NCM811|P_15_Z_60_PEDF|Li shows a worse specific discharge capacity of 36.2 mAh g^−1^ and is short‐circuited at the 132^nd^ cycle without running at −20°C. Best of all, the NCM811|P_15_Z_30_PEDF|Li demonstrates the discharge capacity of 77.2 mAh g^−1^ and conserves 84.6 mAh g^−1^ after 128 cycles. At ‐15°C, the discharge specific capacity of the battery fluctuates due to the brief power outage of the thermotank. Even when the temperature decreases to −20°C, the specific discharge capacity of NCM811|P_15_Z_30_PEDF|Li remains 60.5 mAh g^−1^. The superior long‐term cycling and low temperature performances of NCM811|P_15_Z_30_PEDF|Li stem from the enhanced adsorption capacity of the ultrathin and dense ZIF‐67 layer for small plasticizers like FAN compared with ZIF‐67‐free P_15_Z_0_PEDF. The ultrathin ZIF‐67 decreases Li^+^ transport resistance and LiF, Li_3_N, Li_x_N_y_ enriched SEI reduces Li^+^ transport energy barrier, which overcomes the sluggish transport kinetics at fast charging and low temperature conditions with the help of low solvation energy, low Li^+^ transport energy barrier, and small solvent size of FAN [59]. Meanwhile, the excessively thick and dense ZIF‐67 layer in P_15_Z_60_PEDF hinders the adsorption of FAN, EC, and DMC and increases the Li^+^ transport resistance, resulting in poor battery performance.

Furthermore, even at a high temperature of 60°C, the NCM811|P_15_Z_30_PEDF|Li shows an initial specific discharge capacity of 185.6 mAh g^−1^ at 0.5 C and keeps the capacity retention of 70.0% after 133 cycles, as shown in Figure 5d. Figure 5e clearly demonstrates that the pouch cell shows an initial specific discharge capacity of 185.1 mAh g^−1^ at 0.1 C and stably cycling for 100 cycles with capacity retention of 82.5%. After that, the LED bulb with“DUT” pattern was successfully lit up by NCM811|P_15_Z_30_PEDF|Li pouch cell even with complete folding and cutting states. Developing lighter and thinner electrolytes is crucial for enhancing the energy density of SSLMBs. Consequently, a series of 8 µm P_8_Z_m_PEDF CSEs with ultrathin ZIF‐67 were applied to fabricate NCM811||Li batteries and operated at 0.5 C under 30°C. Figure 5f shows that the NCM811|P_8_Z_15_PEDF|Li exhibits the highest specific discharge capacity of 163.4 mAh g^−1^ and demonstrates a capacity retention of 70% after 395 cycles. Contrarily, the NCM811||Li batteries using P_8_Z_0_PEDF, P_8_Z_10_PEDF, P_8_Z_20_PEDF demonstrate fluctuated coulombic efficiency before 200 cycles, which demonstrates the unstable charge‐discharge process. The corresponding charge‐discharge voltage profiles are depicted in Figure S22. To comprehensively evaluate the performances of this designed P_15_Z_30_PEDF and P_8_Z_15_PEDF with 420 nm and 250 nm ultrathin ZIF‐67 layer, the comparisons of cycling performances and membrane thickness with previous reported relative electrolytes are depicted in Figure 5g, and the detailed operating conditions are listed in Table S2 [60, 61, 62, 63, 64, 65, 66, 67, 68]. Both the 15 µm P_15_Z_30_PEDF with 420 nm ultrathin ZIF‐67 layer and the 8 µm P_8_Z_15_PEDF with 250 nm ultrathin ZIF‐67 layer make the NCM811||Li batteries demonstrating superior overall performances than the previous works. Hence, the interfacial growth of ultrathin MOFs for light and thin electrolytes design deserves to be of great concern, which fundamentally boosts the ion transport kinetics and increases the energy density.

**FIGURE 6:**
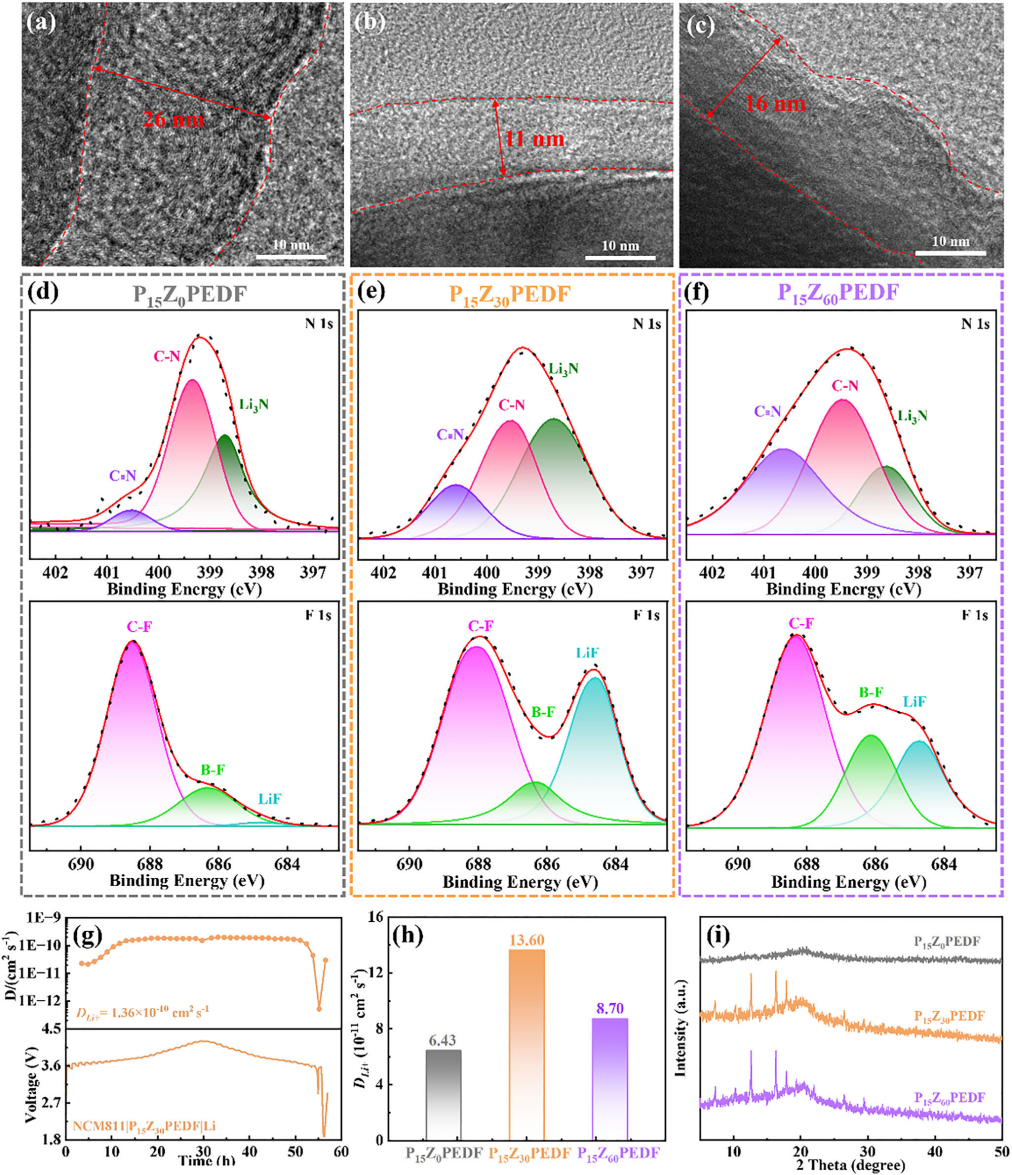
CEI morphology, compositions, and interfacial Li^+^ transport kinetics. TEM images of NCM811 cathode harvested from (a) NCM811|P_15_Z_0_PEDF|Li, (b) NCM811|P_15_Z_30_PEDF|Li, (c) NCM811|P_15_Z_60_PEDF|Li after 20 cycles at 0.5 C; The N 1s and F 1s XPS spectra of cycled NCM811 cathode from (d) NCM811|P_15_Z_0_PEDF|Li, (e) NCM811|P_15_Z_30_PEDF|Li, (f) NCM811|P_15_Z_60_PEDF|Li; The Li^+^ diffusion coefficient measurement by GITT of (g) NCM811|P_15_Z3_0_PEDF|Li, (h) Li^+^ diffusion coefficient, (i) XRD patterns of cycled P_15_Z_0_PEDF, P_15_Z_30_PEDF and P_15_Z_60_PEDF disassembled from NCM811||Li after 20 cycles at 0.5 C.

To further investigate the enhanced antioxidant P_15_Z_30_PEDF towards high‐voltage NCM811 cathode, the morphology and compositions of CEI on cycled NCM811 disassembled from NCM811|P_15_Z_0_PEDF|Li, NCM811|P_15_Z_30_PEDF|Li, and NCM811|P_15_Z_60_PEDF|Li after 20 cycles at 0.5 C were detected by transmission electron microscope (TEM) and XPS. The TEM image in Figure 6a demonstrates the thick CEI of ∼26 nm on the cycled NCM811 cathode based on P_15_Z_0_PEDF|Li, which is due to the severe interfacial side reactions. While the CEI thickness is significantly decreased to ∼11 nm when using P_15_Z_30_PEDF as shown in Figure 6b. The thinner CEI is attributed to the facilitated Li^+^ transport with low resistance due to the 420 nm ultrathin ZIF‐67 and its confinement for plasticizers (EC, DMC, FAN). The thinner CEI layer is expected to be beneficial for battery performance with a wide temperature range, as it shortens the diffusion path of Li^+^ [69]. But when the thickness of ZIF‐67 is increased to 720 nm in P_15_Z_60_PEDF, the CEI also becomes thicker (∼16 nm) and uneven due to the larger Li^+^ transport resistance (Figure 6c). The compositions of CEI are shown by XPS spectra in Figures 6d‐f and Figure S23. The peaks at 398.7 eV from N 1s spectra and 684.8 eV from F 1s spectra represent the Li_3_N and LiF, respectively. As a result, the inorganic components such as Li_3_N and LiF in the CEI on cycled NCM811, coupled with P_15_Z_30_PEDF exhibit a significant increase compared to those with P_15_Z_0_PEDF and P_15_Z_60_PEDF. Thus, the thin and inorganic abundant CEI layer is formed based on P_15_Z_30_PEDF with 420 nm ultrathin ZIF‐67, which provides a fast interfacial Li^+^ conduction channel to enhance the Li^+^ transport kinetics to improve the antioxidant property [59].

The interfacial Li^+^ transport kinetics were further verified by the Li^+^ diffusion coefficient measured via the galvanostatic intermittent titration technique (GITT) as shown in Figure 6g and Figure S24. To sum up in Figure 6h, NCM811|P_15_Z_30_PEDF|Li battery demonstrates higher apparent Li^+^ diffusion coefficient of 1.36 × 10^−10^ cm^2^ s^−1^ than that of NCM811|P_15_Z_0_PEDF|Li and NCM811|P_15_Z_60_PEDF|Li with 6.43 × 10^−11^ cm^2^ s^−1^ and 8.70 × 10^−11^ cm^2^ s^−1^, respectively. Consequently, the lower interfacial Li^+^ conduction barrier between P_15_Z_30_PEDF and NCM811 facilitates rapid Li^+^ transport at the interface. The sequence of Li^+^ diffusion coefficient was consistent well with the conclusion drawn by TEM images and XPS analysis of CEI layer in cycled NCM811 cathode. To verify the stability of the ultrathin ZIF‐67 layer during the battery cycling process, the P_15_Z_0_PEDF, P_15_Z_30_PEDF, and P_15_Z_60_PEDF CSEs disassembled from the aforementioned cycled batteries were detected by XRD, as shown in Figure 6i. The characteristic peaks of P_15_Z_30_PEDF and P_15_Z_60_PEDF are consistent with the specific peaks of ZIF‐67, which indicates that the ultrathin ZIF‐67 layer remains stable during the battery cycling process. Furthermore, as depicted in the SEM morphology of the membrane after cycling (Figure S25), the ZIF‐67 layer is still clearly present in the surface and cross‐sectional images of the electrolyte membrane, demonstrating that ZIF‐67 remains stable after cycling. XPS analysis of the P_15_Z_30_PEDF membrane before and after cycling (Figure S26) reveals no shift in the Co 2p peak position, further confirming the stable existence of ZIF‐67 [70]. Therefore, the interfacial growth of ultrathin MOFs is essential to the ion transport kinetics and stable interfacial compatibility between electrolytes and cathodes, which is beneficial for the battery performance with a wide temperature range and energy density enhancement.

## 3 Conclusion

In summary, the thickness and morphology of MOFs layer are precisely controlled by regulating the reaction time via the controllable permeation interfacial growth method. The thickness of MOFs layer could be decreased to 250 nm and 420 nm with the morphology variation from loose MOFs nanoparticles to a dense MOFs layer. The in‐situ polymerized CSEs in the ultrathin and dense ZIF‐67 based membrane reduce Li^+^ transport resistance and promote lithium salts, thereby increasing the ionic conductivity to 2.70 mS cm^−1^ at 30°C and the Li^+^ transference number to 0.71. Meanwhile, the Li deposition behavior and interfacial Li^+^ transport kinetics are also regulated via ultrathin ZIF‐67 with an enhanced Li^+^ diffusion coefficient and uniform Li^+^ flux. The ultrathin and dense ZIF‐67 balances the trade‐off between ionic conductivity and Li dendrite suppression, ultimately enabling stable long‐term cycling performance of Li||Li symmetric cell for 1000 h at 0.1 mA cm^−2^. The NCM811||Li maintains 70% capacity retention after 446 cycles and shows its feasibility for operation at a wide range of temperatures (−20°C–60°C). The pouch cell exhibits excellent cycling stability and lights up the LED even after being completely folded and cut. This work inspires the structure design of ultrathin MOF‐layer‐based CSEs with high ion conductivity and low interfacial conduction resistance.

## Author Contributions

**Shichen Zhang**: data curation, writing – original draft. **Gaohong He**: resources, writing, review and editing, and funding acquisition. **Ziyi Xin**: methodology, data curation. **Yuchen He**: investigation. **Mengjuan Li**: investigation. **Zhaokai Rui**: investigation, writing – original draft, data curation. **Lu Gao**: investigation. **Xiaobin Jiang**: conceptualization, methodology. **Yihang Li**: methodology, data curation. **Xiangcun Li**: conceptualization, methodology. **Xinhong Qi**: conceptualization, methodology, data curation, investigation, writing – review and editing, writing – original draft and funding acquisition.

## Conflicts of Interest

The authors declare no conflict of interest.

## Supporting information




**Supporting File**: advs76317‐sup‐0001‐SuppMat.docx.

## Data Availability

The data that support the findings of this study are available from the corresponding author upon reasonable request.
