# Supplementary material for: Interfacial Growth of 420 nm Ultrathin and Dense MOFs for Composite Electrolyte to Reduce Li+ Conduction Resistance and Inhibit Lithium Dendrite
Source: Adv Sci (Weinh). 2026 Jul 6;13(53):e76317. doi: 10.1002/advs.76317 (PMC13335458; doi:10.1002/advs.76317)
Supplement: Supplementary file 1 [file ADVS-13-e76317-s001.docx]

**Supporting Information**

**Interfacial growth of 420 nm ultrathin and dense MOFs for composite electrolyte to reduce Li^+^ conduction resistance and inhibit lithium dendrite**

Xinhong Qi, Zhaokai Rui, Shichen Zhang, Yihang Li, Ziyi Xin, Mengjuan Li, Lu Gao, Yuchen He, Xiaobin Jiang, Xiangcun Li, Gaohong He^*^

*State Key Laboratory of Fine Chemicals, Frontiers Science Center for Smart Materials Oriented Chemical Engineering, School of Chemical Engineering, Dalian University of Technology, Dalian 116024, China*

* E-mail: [hgaohong@dlut.edu.cn](mailto:hgaohong@dlut.edu.cn)

**Experimental methods**

1.1 ***Materials***

All chemicals were used as received without further purification. The 2-Methylimidazole (2-MIm, ≥98 %), Polyvinylidene fluoride-co-hexafluoropropylene (PVDF-HFP, m_w_~455000), Dimethylformamide (DMF, ≥99 %), Cobalt nitrate hexahydrate (Co(NO_3_)_2_·6H_2_O), Lithium difluoro(oxalato)borate (LiDFOB, ≥99 %), Lithium bis(fluorosulfonyl)imide (LiTFSI, ≥99 %), Fluoroacetonitrile (FAN, ≥99 %), Poly (ethylene glycol) diacrylate (PEGDA, m_w_=600), and 2,2’-Azobis (2-methylpropionitrile) (AIBN, ≥99 %) were purchased from Aladdin. The Ethylene carbonate (EC, ≥99.99 %) and Dimethyl carbonate (DMC, ≥99.99 %), Polyvinylidene fluoride (PVDF, m_w_~1100000), stainless-steel plate (Φ16 mm), copper plate (Φ16 mm), lithium metal plate (Φ16 mm), CR2025 coin cell case, conductive carbon black coated aluminum foil, lithium-copper foil (thickness of lithium 20 μm), LiNi_0.8_Co_0.1_Mn_0.1_O_2_ (NCM811, single crystal) and N-methyl pyrrolidone (NMP, ≥99.9 %) were obtained from Canrd. The anhydrous methanol and anhydrous ethanol were acquired from Tianjin DaMao. The conductive carbon black (Super P) was supplied by Cyber electrochemical materials.

1.2 ***Synthesis of PVDF-HFP@2-MIm membrane***

The PVDF-HFP@2-MIm membrane was prepared by phase inversion method. 2.0 g of 2-MIm was dissolved in 16.0 g of DMF, followed by adding 4.0 g of PVDF-HFP, and the mixture was stirring vigorously in the glass bottle at 35 °C for 6 h. After that, the solution was ultrasonic to defoam before spreading evenly on the glass plate by membrane scraper (thickness of scraper was 50 μm and 25 μm). The glass plate was then transferred into deionized water bath and phase inverted for 10 min. After dried in 70 °C oven for 24 h, the brown PVDF-HFP@2-MIm membrane was finally obtained, and the thickness of membrane prepared with 50 μm, 25 μm scraper were 15 μm and 8 μm, respectively.

***1.3 Synthesis of P_n_Z_m_ composite membrane with ultrathin MOF layer***

The ultrathin ZIF-67 on PVDF-HFP membrane was prepared through interfacial growth method. To start with, Co(NO_3_)_2_·6H_2_O was dissolved in 100 mL anhydrous methanol followed by vigorously stirring to prepare Co(NO_3_)_2_ solution (0.08 M). Meanwhile, the 0.64 M 2-MIm solution was prepared by dissolving 21.02 g of 2-MIm in 400 mL methanol. After that, the PVDF-HFP@2-MIm membrane was immersed in Co(NO_3_)_2_ solution for 30 min and washed by methanol. The membrane was putted onto the reactor that filled with 2-MIm solution with smooth face upside, and the Co(NO_3_)_2_ was then drop wise added on the membrane. The reaction lasted for 10 min - 60 min in 30 °C thermotank. The P_n_Z_m_ composite membranes were finally obtained after ultrasonic wash with methanol for three times followed by drying in 70 °C oven for 24 h, where *n* means the thickness of membrane in μm and *m* represents the interfacial growth time in min.

***1.4 Preparation of NCM811 cathode***

1.6 g NCM811, 0.2 g PVDF and 0.2 g conductive carbon black were mixed followed by vigorous grinding for 15 min, the mixture was then dispersed into 5.6 mL NMP to obtain the cathode slurry. After that, the mixed slurry was coated on conductive carbon black coated aluminum foil with 100 μm scraper. The NCM811 cathode was finally obtained after drying in 80 °C oven for 24 h.

***1.5 Preparation of P_n_Z_m_PEDF composite solid electrolytes (CSEs)***

The CSEs were prepared in the glove box. Initially, LiTFSI, LiDFOB, PEGDA, EC/DMC (molar ratio of 1:1) mixture and FAN with mass ratio of 3:7:10:50:2.5 were sufficiently blended for 4 h. After that, the thermal initiator AIBN (1.5 wt% of PEGDA) was added into the mixture with vigorous stirring for 20 min to obtain the electrolyte precursor. The P_n_Z_m_PEDF CSEs were finally obtained after in-situ polymerization of electrolyte precursor in P_n_Z_m_ membrane by heating in 70 °C oven for 2 h.

***1.6 Fabrication of coin and pouch cells***

The coin and pouch cells were assembled in the glove box as well. The SS||SS (stainless steel), SS||Li, Li||Li, Li||Cu and NCM811||Li coin cells were assembled for electrochemical tests.

**Coin cell:** At first, the P_n_Z_m_ membrane and NCM811 cathode was tailored into Φ19 mm and Φ12 mm by precision disk cutting machine (MSK-T10), respectively. After that, the cathode plate, P_n_Z_m_ membrane, anode plate, SS plate and spring plate were put into the CR2025 case in sequence, during which the electrolyte precursor was dripped on each side of the P_n_Z_m_ membrane. The coin cells were finally obtained after compacting by hydraulic crimping machine (MSK-110) and in-situ polymerization in 70 °C oven for 2 h.

**Pouch cell:** To start with, the NCM811 cathode foil was tailored into 43×56 mm and the lithium loaded copper foil were tailored into 45×58 mm through the electrode punching machine (MSK-180-S). After that, the aluminum tabs and nickel tabs were ultrasonic welded on cathode and anode by ultrasonic punching machine (MSK-800), respectively. The electrolyte precursor was then dripped on P_15_Z_30_ membrane followed by packing the cell with aluminum-plastic film with vacuum sealing machine. The pouch cell was finally obtained after 2 h heating in 70 °C oven, and the detailed parameters were listed in Table S1.

***1.7 Materials characterizations***

The scanning electron microscopy (SEM, SU5000) was adopted to observe the morphology of P_n_Z_m_ membrane and lithium metal anode surface after cycling. The X-ray diffraction (XRD) was detected by multifunctional X-ray diffractometer (SmartLab 9kw). The polymerization behavior was detected by advanced Fourier transform infrared spectrometer (FTIR, iS50). The thermogravimetric analysis for CSEs were examined to analyze thermal stability through thermogravimetric instrument (TGA Instrunments-Waters LLC, TGAQ50) in N_2_ atmosphere. The Raman spectra were detected by Raman spectroscopy (inVia Qontor, Renishaw). The solid-state magic angle spinning nuclear magnetic resonance (MAS NMR, ^7^Li spectra) of CSEs were detected by solid state high power nuclear magnetic resonance spectrometer (Agilent DD2-500MHz). The Young’s modules of electrolytes were characterized by atomic force microscope (AFM) (Dimension FastScan, Bruker). The components of solid electrolyte interphase (SEI) and cathode-electrolyte interphase (CEI) were characterized through X-ray photoelectron spectroscopy (XPS, Axis Supra+). The SEI components in depth were detected by time-of-flight secondary ion mass spectrometry (TOF-SIMS, M6 Hybrid). The CEI morphology was observed by Cryo transmission electron microscope (TEM, JEM-F200(CF-CR)).

***1.8 Electrochemical performance measurements***

The ionic conductivity, lithium-ion transference number, exchange current density, electrochemical stability window, Li||Li interface impedance and activation energy were analyzed through serious of tests on electrochemical workstation (Princeton applied research, AMETEK, PMC 1000).

The ionic conductivity was calculated through electrochemical impedance spectroscopy (EIS) test of SS|P_n_Z_m_PEDF|SS symmetric cells while the activation energy (*E_a_*) was calculated by fitting Arrhenius formula through impedance under temperature gradient from 30 °C to 60 °C with 5 °C as gradient. Specifically, the frequency range was from 10^6^ Hz to 1 Hz with amplitude of 10 mV, and three plots was tested under one frequency with high stability test mode. The ionic conductivity (*σ*) under various temperatures were calculated through equation (1), while *E_a_* was calculated by Arrhenius equation (2).

$\sigma=\frac{L}{R\times S}$	(1)

Where *σ* represents the ionic conductivity, *L* is the thickness of CSE, *R* represents the impedance of CSE, and *S* means the effective contact area.

$\sigma=Ae^{(\frac{-Ea}{kT})}$	(2)

Where *A* represents pre-exponential factor, *e* is the natural logarithm base, *k* and *T* were Boltzmann constant and Kelvin temperature, respectively.

The Li||Li cell was used to examine the exchange current density by Tafel curves, and SS||Li cell was employed to examine the electrochemical stability window by linear scanning voltammetry (LSV) curves. To be specific, the scanning speed of 0.1 mV s^–1^ from initial potential to 6 V on SS|P_n_Z_m_PEDF|Li battery was applied for stable linear scanning voltammetry (LSV) measurement. The lithium-ion transference number (*t*_Li+_) was calculated through applying constant potential polarization on Li||Li symmetric cells. A step voltage of 0.01 V (*∆V*) was applied on Li||Li symmetric cells to obtain direct current (DC) polarization curve, and the impedance of Li||Li cells before and after polarization were tested by EIS as well. Based on EIS and DC polarization curves, the *t*_Li+_ can be calculated through Bruce-Vincent equation (3):

$t_{Li+}=\frac{I_{ss}(\Delta V-I_{0}R_{0})}{I_{0}(\Delta V-I_{ss}R_{ss})}$	(3)

Where *I*_0_ and *I*_ss_ were initial and stable currents, while *R*_0_ and *R*_ss_ represent resistances before and after polarization, respectively.

The Li||Li symmetric cell was also applied to identify interface activation energy. To be specific, the EIS curves of Li||Li cells with frequency range from 10^5^ Hz to 0.1 Hz and amplitude of 10 mV under temperature gradient from 30 °C to 80 °C with 10 °C as gradient were obtained. The interface impedance (*R_i_*) was obtained through relaxation time distribution (DRT), and the interface activation energy can be calculated by equation (4):

$\frac{1}{R_{i}}=Ae^{(\frac{-Ea}{kT})}$	(4)

The Li||Li, Li||Cu and NCM811||Li were galvanostatic charged and discharged at different current densities and different temperatures to obtain coulombic efficiency, Li^+^ diffusion coefficient, long-term cycling performances and rate performances on LAND battery testing system (LANHE, CT3002A).

The coulombic efficiency (CE) of Li plating/stripping was measured by the Aurbach method. The Li||Cu cells were plated/stripped at a current density of 0.1 mA cm^–2^ after pre-lithiation at 1 mAh cm^–2^, and finally stripping to 1.0 V (vs. Li^+^/Li). The value of CE was calculated by the Equation (5):

$CE= \frac{nQ_{c}+Q_{s}}{nQ_{c}+Q_{T}}$	(5)

Where 𝑄*_S_* and 𝑄*_T_* are stripping capacity and plating capacity, respectively. 𝑄_𝑐_ is the plating/stripping capacity of each cycle (here 𝑄_𝑐_=200 μAh), and 𝑛 stands for the cycle number (here *n*=19).

**Supplementary Figures**

**Figure S1.** The schematic illustration of self-made membrane preparation device.

**Figure S2. Cross-sectional EDS mapping of P_15_Z_30_.**

**Figure S3. Cross-sectional TEM images of the P_15_Z_30_.**

**Figure S5.** Photographs of (a) liquid electrolyte precursor before polymerization and (b-c) solid-state electrolyte after polymerization.

**Figure S6.** FTIR spectra of liquid electrolyte precursor and P_15_Z_m_PEDF CSEs.

**Figure S7.** TGA curves of P_15_Z_m_PEDF CSEs.

**Figure S8.** Nyquist curves of SS||SS cells with (a) P_15_Z_0_PEDF, (b) P_15_Z_15_PEDF, (c) P_15_Z_30_PEDF, (d) P_15_Z_45_PEDF and (e) P_15_Z_60_PEDF as electrolytes from 30 ^o^C to 60 ^o^C.

**Figure S9. Ionic conductivity and EIS impedance of P_8_Z_m_PEDF composite solid electrolyte.**

**Figure S10.** The direct current polarization curves with EIS curves before and after polarization of (a) Li|P_15_Z_0_PEDF|Li, (b) Li|P_15_Z_15_PEDF|Li, (c) Li|P_15_Z_45_PEDF|Li and (d) Li|P_15_Z_60_PEDF|Li.

**Figure S11.** The pie graph illustration of Raman spectra peak area ratio (a) P_15_Z_0_PEDF, (b) P_15_Z_30_PEDF, (c) P_15_Z_60_PEDF.

**Figure S12.** **Cycling performance of Li|P_15_Z_m_PEDF|Li at different current densities (a) 0.2 mA cm^-2^, (b) 0.3 mA cm^-2^, (c) Critical current density of Li|P_15_Z_m_PEDF|Li.**

**Figure S13.** Nucleation overpotentials and coulombic efficiencies of Li||Cu cells using (a) P_15_Z_0_PEDF and (b) P_15_Z_60_PEDF.

**Figure S14.** The Young’s modulus of (a) P_15_Z_0_PEDF, (b) P_15_Z_30_PEDF, (c) P_15_Z_60_PEDF.

**Figure S15.** The EIS profiles before cycling and after 10 h, 20 h cycling at 0.1 mA cm^−2^ for (a) Li|P_15_Z_0_PEDF|Li, (b) Li|P_15_Z_30_PEDF|Li and (c) Li|P_15_Z_60_PEDF|Li.

**Figure S16.** The deep etching XPS spectra of C 1s for LMA disassembled from Li||Li symmetric cells after 50 h cycling at 0.1 mA cm^–2^ using (a) P_15_Z_0_PEDF, (b) P_15_Z_30_PEDF, (c) P_15_Z_60_PEDF.

**Figure S17.** The deep etching XPS spectra of F 1s for LMA disassembled from Li||Li symmetric cells after 50 h cycling at 0.1 mA cm^–2^ using (a) P_15_Z_0_PEDF, (b) P_15_Z_30_PEDF, (c) P_15_Z_60_PEDF.

**Figure S18.** The deep etching XPS spectra of N 1s for LMA disassembled from Li||Li symmetric cells after 50 h cycling at 0.1 mA cm^–2^ using (a) P_15_Z_0_PEDF, (b) P_15_Z_30_PEDF, (c) P_15_Z_60_PEDF.

**Figure S19.** TOF-SIMS depth profiles of LiF_2_^−^ and Li_2_N^−^ for LMA disassembled from Li||Li symmetric cells after 50 h cycling at 0.1 mA cm^–2^ using P_15_Z_0_PEDF, P_15_Z_30_PEDF, P_15_Z_60_PEDF.

**Figure S20.** Cycling performances of Li|P_8_Z_0_PEDF|Li, Li|P_8_Z_10_PEDF|Li, Li|P_8_Z_15_PEDF|Li and Li|P_8_Z_20_PEDF|Li symmetric cells at 0.1 mA cm^–2^.

**Figure S21.** Charge-discharge voltage profiles at different cycling numbers of (a) NCM811|P_15_Z_0_PEDF|Li, (b) NCM811|P_15_Z_30_PEDF|Li, (c) NCM811|P_15_Z_60_PEDF|Li.

**Figure S22.** Charge-discharge voltage profiles at different cycling numbers of (a) NCM811|P_8_Z_0_PEDF|Li, (b) NCM811|P_8_Z_10_PEDF|Li, (c) NCM811|P_8_Z_15_PEDF|Li, (d) NCM811|P_8_Z_20_PEDF|Li.

**Figure S23.** The C 1s XPS spectra of NCM811 cathode harvested from NCM811|P_15_Z_0_PEDF|Li, (b) NCM811|P_15_Z_30_PEDF|Li, (c) NCM811|P_15_Z_60_PEDF|Li after 20 cycles at 0.5 C.

**Figure S24.** The Li^+^ diffusion coefficient measurement with galvanostatic intermittent titration technique (GITT). (a) NCM811|P_15_Z_0_PEDF|Li, (b) NCM811|P_15_Z_60_PEDF|Li.

**Figure S25. The SEM of membrane disassembled from Li|P_15_Z_30_PEDF|Li after 50 h cycling at 0.1 mA cm**^–1^ **(a) surface, (b) cross-section.**

**Figure S26.** The XPS spectra of the P_15_Z_30_PEDF disassembled from the Li|P_15_Z_30_PEDF|Li battery after 50 h cycling at a current density of 0.1 mA cm^–1^: (a) after cycling, (b) before cycling.

**Supplementary Tables**

Table S1. The specifications of the LFP||LFP pouch cell

| **Cell component** | **Specification** | **Parameter** |
| --- | --- | --- |
| Cathode (LFP with Al current collector) | LFP ratio | 0.8 |
|  | Active material loading | 1.38 mg cm^-2^ |
|  | Size | 45*58 mm |
|  | Theoretical specific capacity | 6.12 mAh |
| Anode (Li with Cu current collector) | Thickness (Li) | 50 μm |
|  | Thickness (Cu) | 8μm |
|  | Size | 45*58 mm |
|  | Theoretical specific capacity | 269 mAh |
| Full cell | Voltage range | 2.5 V-4.0 V |
|  | Real specific capacity | 153.4 mAh g^-1^ (0.1 C) |
|  |  | 149 mAh g^-1^ (0.2 C) |
|  | Total capacity | 5.53 mAh (0.1 C) |
|  |  | 5.37 mAh (0.2 C) |

Table S2. Specific parameters of performance comparison with other works

| Cathode  material | Membrane thickness (μm) | Rate (C) | Cycle number | Capacity retention (%) | This  Work (%) | Ref |
| --- | --- | --- | --- | --- | --- | --- |
| NCM811 | 70 | 0.5 | 300 | 73 | 79.1 | [59] |
| NCM811 | 83 | 0.5 | 150 | 74 | 88.8 | [60] |
| NCM811 | 49 | 0.5 | 400 | 47.1 | 73.3 | [61] |
| NCM811 | 100 | 0.5 | 30 | 85 | 97.9 | [62] |
| NCM811 | 13 | 0.2 | 70 | 94.4 | 94.7 | [63] |
| NCM811 | 52 | 0.5 | 150 | 80.4 | 88.8 | [64] |
| NCM811 | 212 | 0.2 | 100 | 78 | 92.4 | [65] |
| NCM811 | 57 | 0.5 | 100 | 83 | 92.4 | [66] |
| NCM811 | 300 | 0.2 | 100 | 92 | 92.4 | [67] |
